# Genetic conflict outweighs heterogametic incompatibility in the mouse hybrid zone?

**DOI:** 10.1186/1471-2148-8-271

**Published:** 2008-10-03

**Authors:** Miloš Macholán, Stuart JE Baird, Pavel Munclinger, Petra Dufková, Barbora Bímová, Jaroslav Piálek

**Affiliations:** 1 Laboratory of Mammalian Evolutionary Genetics, Institute of Animal Physiology and Genetics, Academy of Sciences of the Czech Republic (ASCR), Brno, Czech Republic; 2 Institute of Botany and Zoology, Masaryk University, Brno, Czech Republic; 3 INRA, Centre de Biologie et de Gestion des Populations, Campus International de Baillarguet, Montferrier-sur-Lez, France; 4 CIBIO, Centro de Investigação em Biodiversidade e Recursos Genéticos, Campus Agrário de Vairão, Vairão, Portugal; 5 Biodiversity Research Group, Department of Zoology, Faculty of Science, Charles University, Prague, Czech Republic; 6 Department of Genetics, University of South Bohemia, Èeské Budìjovice, Czech Republic; 7 Department of Population Biology, Institute of Vertebrate Biology, ASCR, Studenec, Czech Republic

## Abstract

**Background:**

The *Mus musculus musculus/M. m. domesticus *contact zone in Europe is characterised by sharp frequency discontinuities for sex chromosome markers at the centre of wider clines in allozyme frequencies.

**Results:**

We identify a triangular area (approximately 330 km^2^) where the *musculus *Y chromosome introgresses across this front for up to 22 km into *domesticus *territory. Introgression of the Y chromosome is accompanied by a perturbation of the census sex ratio: the sex ratio is significantly female biased in *musculus *localities and *domesticus *localities lacking Y chromosome introgression. In contrast, where the *musculus *Y is detected in *domesticus *localities, the sex ratio is close to parity, and significantly different from both classes of female biased localities. The geographic position of an abrupt cline in an X chromosome marker, and autosomal clines centred on the same position, seem unaffected by the *musculus *Y introgression.

**Conclusion:**

We conclude that sex ratio distortion is playing a role in the geographic separation of speciation genes in this section of the mouse hybrid zone. We suggest that clines for genes involved in sex-ratio distortion have escaped from the centre of the mouse hybrid zone, causing a decay in the barrier to gene flow between the two house mouse taxa.

## Background

Increasingly, evidence suggests that the sex chromosomes harbour more genes causing disruption of fertility and/or viability in hybrids than autosomes and hence will be under stronger selection in hybrid zones [1-8]. While the causes of this disruption have generally been assumed to follow the Dobzhansky-Muller model of accumulation of incompatibilities [8], genes involved in genetic conflicts have also been implicated [9-11]. In either case, gene flow of sex-linked markers across a hybrid zone is expected to be impeded, resulting in abrupt clines [12], the best examples of which have been reported from the contact zone between two house mouse subspecies, *Mus musculus musculus *and *M. m. domesticus *(see [13] for review).

**Figure 1:**
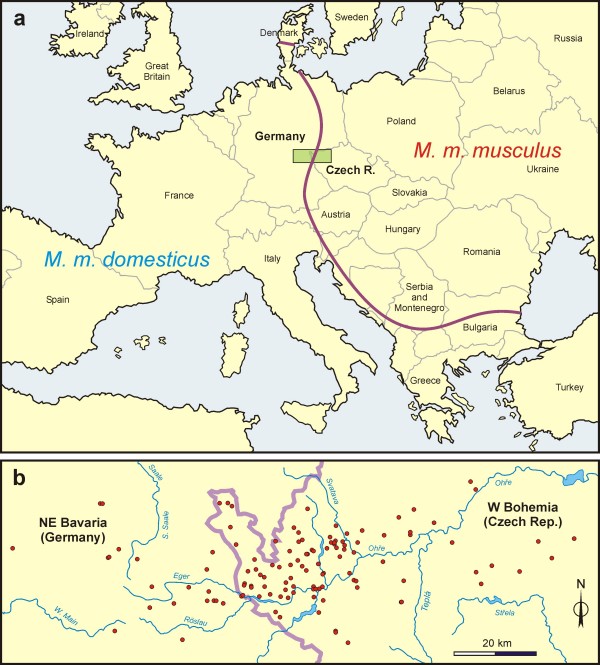
**The *M. m. musculus*/*M. m. domesticus *hybrid zone in Europe**. (a) The course of the zone is shown as bold line and the location of the study area as shaded rectangle. (b) The detail of the study area with sampling sites indicated.

The *musculus-domesticus *hybrid zone crosses the Jutland peninsula in Denmark and runs from the Baltic coast in East Holstein (northern Germany) across central Europe and the Balkans to the Black Sea (Figure 1; see also [14] and references therein). The transition of X-chromosome markers across various portions of this zone have been shown to be steep in comparison with autosomal markers (Bulgaria: [15]; southern Germany: [16,17]; Denmark: [18]; central Europe: [14]). Similarly, virtually no introgression was found for the Y chromosome in Bulgaria [19], northern Germany [20], and Denmark [21,22]. These observations have built up a remarkably consistent picture of wider autosomal clines *versus *more narrow sex linked clines spanning more than 2500 kilometres of the contact front between the subspecies in Europe, remarkable because it seems the dynamics of secondary contact are broadly similar over a very large geographic range relative to the dispersal of house mice [14,23]. This geographic repeatability and the fact that a model organism is involved, gives the *musculus-domesticus *contact zone the potential to play a central role in understanding the general features of secondary contact and the nature of barriers to gene flow between taxa, and in particular the action and nature of 'speciation' genes associated with sex chromosomes. However, a survey focused on the distribution of autosomal and sex-limited diagnostic markers across the Czech and Slovak Republics revealed an unexpectedly gradual transition of the Y chromosome compared to an X locus, and even to five of the autosomal loci analyzed [24], contradicting the results from other parts of the hybrid zone and causing us to question the generality of our understanding of the outcome of house mouse contact.

The history of initial contact between *M. m. musculus *and *M. m. domesticus *is unclear. It has been suggested that source populations first met in the southern region of the current hybrid zone, and only more recently in central and northern Europe [25-27], with progressive contact from south to north similar to a zipper being pulled up through Europe. Secondary contact along such a front would initially produce parallel clines similar in width (i.e., concordant) and position (i.e., coincident). However, concordance would degenerate as clines at neutral loci widen compared to loci experiencing a barrier to gene flow [12]. In contrast, coincidence between cline centres is expected to be maintained by selection against hybrids. A tension zone [28], where selection against hybrids arises due to genetic rather than environmental factors, is not tied to a particular geographic area, and differential gene flow from the source regions towards a central hybrid sink will naturally push the zone toward troughs in population density and barriers in the environment. In the same way, clines at selected loci, each of which can be thought of as a barrier to gene flow, will be pushed by differential pressure of population density to coincide and their association is expected to form sharp steps at coincident cline centres [12]. In general, hybrid zone studies identify a set of loci with coincident cline centres, and an occasional outlier locus (or a few loci) with a displaced cline [29,30]. In cases of contact across a long front, such as the *musculus-domesticus *zone in Europe, a description of a set of loci with coincident cline centres forms the basis for further analysis. The consensus centre is used to map the path of the front, which may curve across the landscape at different geographic scales (cf *Chorthippus *grasshoppers: [31]; *Bombina *toads: [32]). This line is the geographic zero from which all distances of sampling localities are measured in order to estimate cline widths. However, if clines are not parallel the width of a non-coincident cline will be systematically overestimated and this is a potential explanation of the apparently gradual change of Y chromosome frequencies found by Munclinger et al. [24]: that the Y chromosome front is in fact following a different path from the consensus. It is not immediately clear how to estimate the positions of cline centres along a front allowing for this possibility since estimates of cline position depend on the model of cline cross-section (shape) assumed.

As a first approach it seems judicious to focus on inferring the path of the displaced cline while making as few assumptions as possible about its shape. Here we analyze the orientation of Y chromosome change in the Czech-Bavarian section of the *musculus*-*domesticus *hybrid zone assuming only that the cross-section of its cline is monotonic and that at the scale of the field sampling, its front is approximately linear. We present for the first time strong evidence that its orientation is strikingly different from the consensus contact front. Then we show that, when it is analyzed with respect to its own orientation rather than that of the consensus front, the Y chromosome cline width estimate is significantly reduced, and of the same order of magnitude as in other portions of the *musculus-domesticus *hybrid zone in Europe. We also map the geographic region where the path of the Y chromosome departs from the consensus front, describing a triangular salient of *musculus *Y chromosome introgression pushing far into *domesticus *territory. Pooling all census data from the *musculus *side of the zone, we demonstrate a significant female bias in the census sex ratio. A similar and significant female bias is also found in those *domesticus *localities without introgressed Y chromosomes. In contrast, *domesticus *localities where the *musculus *Y has introgressed show a sex ratio close to parity, the proportion of males being significantly higher here than in either of the other locality classes. We note that the mitochondrial genome displays a geographic pattern of introgression similar to the Y chromosome, though weaker and more stochastic. We discuss the relationship between the Y chromosome invasion and the perturbation of the sex ratio and suggest the Y invasion is due to manipulation of the sex ratio in its favour. We point out that this manipulation need not have been "selfish": male favouring factors would increase by natural selection when countering the selfish action of female favouring factors. Finally, we consider the possibility that the spatial pattern of the mitochondrial marker, similar to that of the Y, may be a trace of a previous introgression from *musculus *to *domesticus*, associated with the spread of such a female favouring element. If this is the case, the Y chromosome introgression is not the first breakdown of the consensus hybrid zone associated with perturbation of the sex ratio, but rather the current incursion of an *ongoing *arms race between elements distorting the sex ratio.

## Results

### Allele frequencies and the orientation of change across the field area

**Figure 2:**
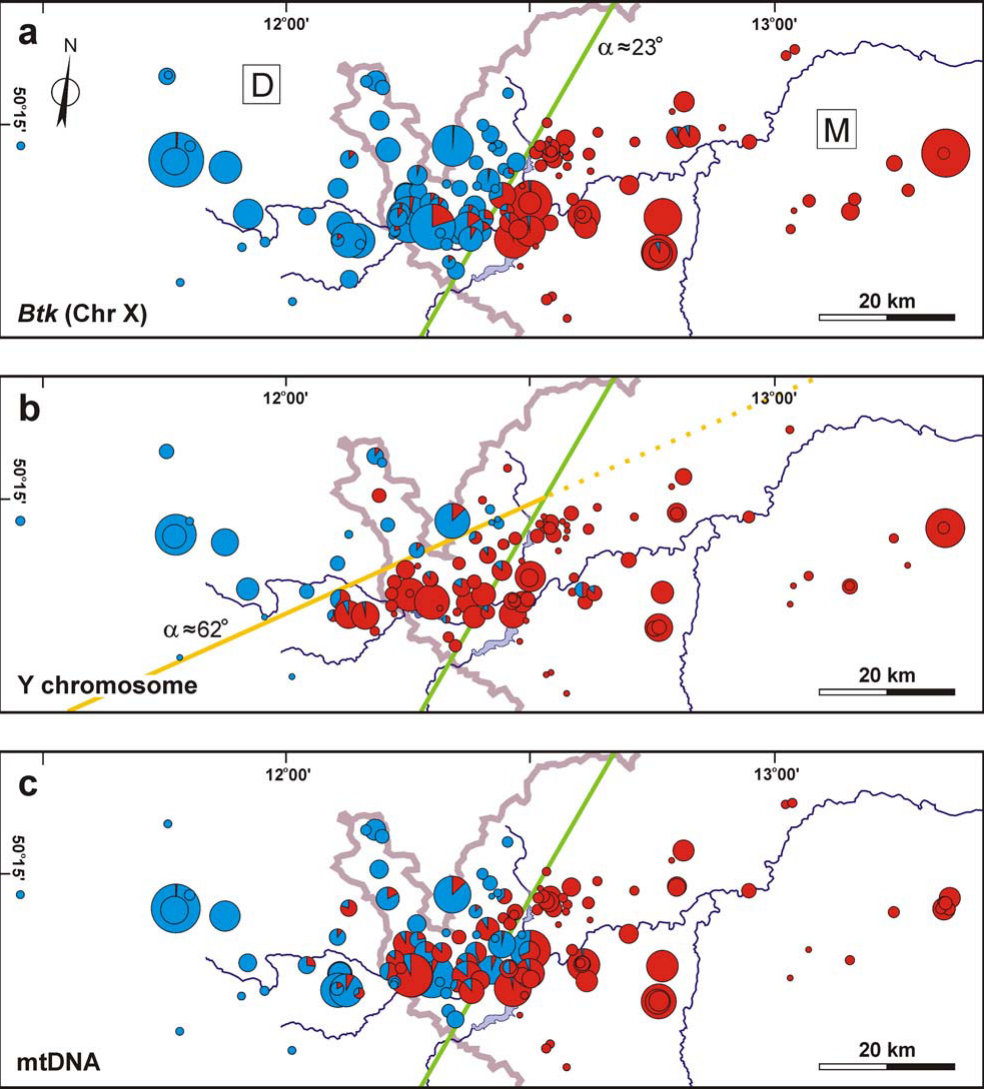
**Allele frequencies across the field area**. Frequencies of *musculus *(red) and *domesticus *(blue) alleles are shown for the X chromosome marker (a), Y chromosome (b), and mtDNA (c). The pies are proportional to sample sizes. The bold lines indicate orientation of the X and Y fronts, respectively, estimated using the PAVA algorithm; the X marker orientation is almost identical to that of the consensus over all loci (see Table 1). M and D denote *musculus *and *domesticus *regions delimited by the green line.

**Figure 3:**
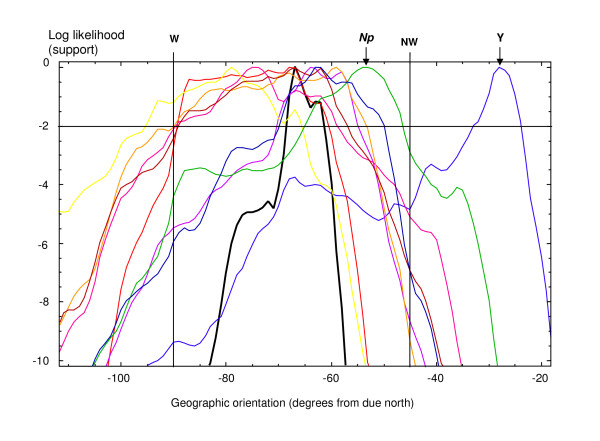
**Likelihood (support) profiles for monotonic change in allele frequencies**. The profiles show likelihood of monotonic change of individual markers from *M. m. musculus *to *M. m. domesticus*, moving through the field area following a compass bearing in a 90° range including due west (W) and northwest (NW). Yellow: *Gpd1*; pink: *Abpa*; red: *Btk *(X chromosome); brown: *Mpi*; orange: *Idh1*; purple: *Es1*; dark blue:*Sod1*; green: *Np*; blue: Y chromosome; bold black: consensus support over all loci. For clarity of presentation the profiles are smoothed over a window of 3°. Since the smoothing removes precise details around the maxima these may not correspond precisely to Table 1 which is based on the original (unsmoothed) profiles.

Frequencies of *musculus *alleles at the *Btk *locus at all the sampling sites under study are depicted as pie diagrams in Figure 2a. The transition of this X-chromosome marker from the *domesticus *to the *musculus *side is rather abrupt and the position and orientation of its front is very similar to the position and orientation of a consensus over autosomal loci [14]. On the other hand, the spatial distribution of Y chromosome frequencies is very different from that of other markers (Figure 2b). Figure 3 shows log likelihood (support) profiles for monotonic change in allele frequencies from *musculus*-like to *domesticus*-like, moving through the field area following a compass bearing lying within a 90° range including due west (W) and northwest (NW). The Y chromosome is a clear outlier, its most likely change being oriented roughly 45° clockwise of the other loci. Summing log likelihood profiles over all loci produces a consensus profile (in bold black in Figure 3) under the assumption that the orientation of change in allele frequencies is the same for all the loci.

Table 1 shows the maximum likelihood estimate (MLE) and two unit support bounds for the orientation of each locus and the consensus. The latter falls almost exactly on the MLE orientation of the X marker (*Btk*) and within the two unit support bounds for the orientation of every other locus except *Np *and the Y chromosome, while the support intervals for all loci except the Y overlap with that of the consensus. Hence there is strong evidence that the Y differs from the consensus and weak evidence that *Np *may follow a different orientation.

**Table 1:** Maximum-likelihood estimates (MLE) and 2LL-unit support bounds for orientation of change (in degrees from due north) of allele frequencies for individual loci; two consensus orientations were estimated, one summed over all nine loci (Consensus) and the second one excluding *Np *and Y chromosome (see text for details).

LL profile	Orientation estimate and 2LL-unit support			Comparison to consensus*	
	
	MLE	Lower	Upper	ΔLL	*P*
*Gpd1*	-79.7	-94.0	-65.6	1.39	0.09
*Abpa*	-74.3	-90.4	-57.8	0.71	0.23
*Idh1*	-69.7	-88.9	-53.4	0.72	0.23
*Btk *(X chr.)	-67.3	-88.9	-60.4	0.03	0.81
*Sod1*	-64.2	-69.4	-49.9	0.67	0.25
*Es1*	-63.3	-69.7	-55.2	1.12	0.13
*Mpi*	-61.8	-88.7	-57.7	0.17	0.56
*Np*	-54.5	-64.2	-46.0	2.66	0.02*
Y chr.	-28.4	-32.7	-24.2	3.62	0.007**
Consensus	-67.4	-68.3	-63.8		
Consensus*	-67.4	-68.3	-65.6		

Treating both *Np *and the Y chromosome as outliers, we can estimate a reduced consensus (hereafter referred to as consensus*) likelihood profile from the remaining loci, assessing potential effects of their inclusion or exclusion. Measuring change at a locus following the consensus* MLE orientation, rather than its own MLE, causes a drop (ΔLL) in support for monotonic change and the significance (*P*) of these drops can be weighed by comparison of 2ΔLL to the χ^2 ^distribution with one degree of freedom. When significance is judged at the 99% level all non-Y loci can be treated as changing in the consensus* orientation without significant reduction in their likelihood of monotonic change. Furthermore, regardless of inclusion or exclusion of both outliers, the consensus MLE orientation remains the same (cf. consensus and consensus* MLE's in Table 1), i.e., -67.4° from due north.

### The coincidence of cline centres and the consensus front

**Figure 4:**
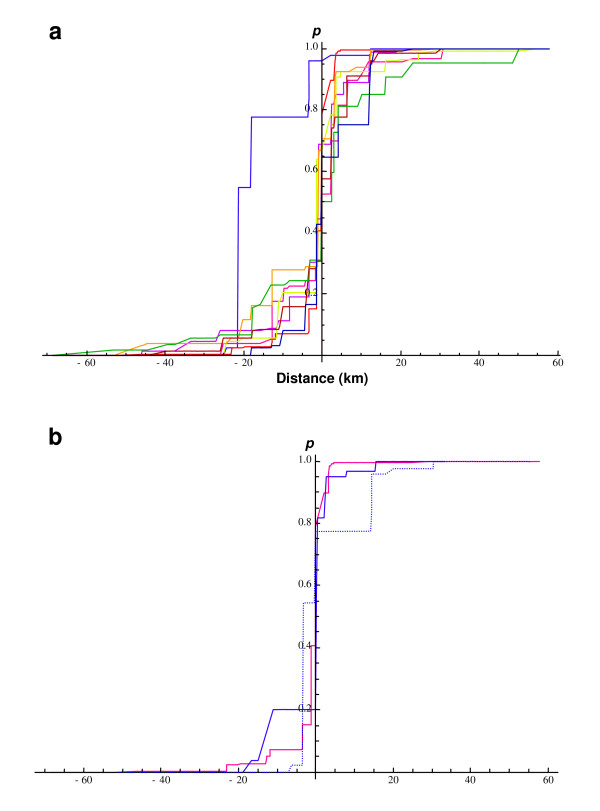
**Monotonic clines for different orientations**. (a) Clines assuming the markers change in the consensus orientation -67.4° from due north. Yellow: *Gpd1*; pink: *Abpa*; red: *Btk *(X chromosome); brown: *Mpi*; orange: *Idh1*; purple: *Es1*; dark blue:*Sod1*; green: *Np*; blue: Y chromosome. (b) Monotonic clines for the X marker (red) and uncorrected Y chromosome (dotted blue) assuming the consensus orientation; solid blue line: the corrected Y chromosome cline assuming the marker changes monotonically along its own most likely orientation of change (-28.4° from due north; cf. Table 1). Note that cline centres have been arbitrarily shifted on the x-axis to allow a direct comparison of cline shapes.

Figure 4a shows the most likely monotonic change in allele frequencies at all eight non-Y loci when clines are traversed with respect to the consensus MLE orientation. While there is a range of cline shapes, this variation revolves round an interval of less than one kilometre, within which all loci pass through 50%. Thus the clines at these loci are highly coincident, and variation in cline width and symmetry forms a bowtie around a knot of coincident centres. For the purposes of illustration, the Y chromosome cline is also shown, as estimated assuming its displacement was parallel to the consensus. While this gives some notion of the scale of Y chromosome introgression (> 20 km), the width and shape of the cline will be misrepresented, because it is not being looked at in perpendicular cross-section. Measuring cline widths using the software Analyse (see Methods), the width of the Y cline along this consensus orientation is estimated at 28.1 km (lower and upper bounds: 19.5–43.5) for the sigmoid model and 19.0 (14.5–25.6) km for the asymmetric stepped model, with little justification for the complicated step hypothesis compared to the simpler sigmoid change.

### The width of X and Y clines

Figure 4b highlights the X and Y clines from Figure 4a, shifting the Y cline (blue, dotted) arbitrarily to the right so that its misrepresented shape can be compared to the X chromosome (red). In addition, the corrected Y cline shape is shown (blue), measured with respect to its own most likely orientation of change (-28.4° from due north). The corrected width is 14.6 (10.5–21.5) km for the sigmoid model whereas the asymmetric stepped cline width falls within the support interval 0.1–6.4 km, with the stepped hypothesis justified in comparison to the sigmoid one (ΔLL = 8.58; df = 4 ; *P *= 0.002). The corrected stepped Y cline fit is significantly better than the uncorrected one (ΔLL = 11.20; df = 1; *P *= 2 × 10^-6^). The corrected Y estimates are of the same magnitude as for the *Btk *locus on the X chromosome: a sigmoid *Btk *is estimated at 9.7 (6.9–16.1), a stepped *Btk *at 4.7 (0.1–8.2) km, and again, the stepped hypothesis is justified in comparison to the sigmoid model (ΔLL = 7.73; df = 4; *P *= 0.006).

### The geography of the zone, Y introgression, and sex ratios

Figure 2a shows the sampling of allele frequencies at the *Btk *locus, and maps the consensus front over this locus and the autosomal loci onto the field area. This front is used to divide the field area into two regions. To the east of the consensus front is the *M. m. musculus *side of the zone (labelled M), while to the west is the *M. m. domesticus *range (labelled D). Figure 2b shows the sampling of allele frequencies at the Y locus and compares the centre of change of the Y cline to that of the remaining loci. Measuring the triangular area between the two fronts allows us to quantify the geographic extent of *musculus *Y introgression. A line departing at right angles from the consensus front (green line) to reach the Y front (yellow line) and passing through the southwestern-most of the localities known to be introgressed, covers a distance of ~22 km, and encloses an area of ~330 km^2^.

Table 2 divides sampling localities into three categories: those in the M region, those in the D region where no Y chromosome introgression has been detected (DY_D_), and those in the D region where the *musculus *Y has been detected (DY_M_). Only localities where at least one Y chromosome has been assayed are included in the analysis.

**Table 2:** Frequency of the *musculus *Y chromosome, hybrid indices (taken as frequencies of *musculus *alleles averaged over eight non-Y loci) in males and females, and the census sex ratio according to category of sampling locality.

	DY_D_	DY_M_	M
*N *(Localities)	20	40	55
			
Y total	187	448	417
Y *musculus*	0	369	412
Freq. Y	0	0.824	0.99
			
Census total	509	1062	1092
			
Males total	226	545	491
Alleles *musculus *(Total)	68(2479)	1343(6682)	5124(5551)
Freq. *musculus*	0.03	0.20	0.92
			
Females total	283	517	601
Alleles *musculus *(Total)	54(2858)	1366(6840)	7059(7546)
Freq. *musculus*	0.02	0.20	0.94
			
Proportion of males	0.44	0.51	0.45
Pr(Bin)	0.0065	0.2037	0.0005

Pr(Bin) stands for the probability that under the binomial distribution we observe a similar or more extreme deviation from 1:1 by chance alone.

There is a clear and significant female bias in the census sex ratio in both the M and DY_D _localities. In contrast, the DY_M _localities show a sex ratio close to parity. Pairwise differences between the categories can be tested by contrasting two hypotheses: H0, two censuses are drawn from populations with the same underlying sex ratio *p*_0_; H1, two censuses are drawn from populations with different underlying sex ratios, *p*_1,A_, *p*_1,B_. In each case the underlying sex ratio is estimated as the proportion of males in the relevant set of observations. This proportion is the maximum likelihood estimate of *p*. The probability of a set of observations, conditioned on an underlying sex ratio, is calculated according to the binomial distribution with parameter *p*. The likelihood of the observations is proportional to this probability. As the hypotheses concern the same set of observations, log likelihoods for the two hypotheses can be compared using a G test with one degree of freedom. The comparisons are as follows: DY_D_| DY_M_: *p*_0 _= 771/1571; *p*_1,A_, *p*_1,B _= 226/509, 545/1062; ΔLL = 3.300; *P *= 0.010. DY_M_| M: *p*_0 _= 518/1077; *p*_1,A_, *p*_1,B _= 491/1092, 545/1062; ΔLL = 4.358; *P *= 0.003. DY_D_| M: *p*_0 _= 717/1601; *p*_1,A_, *p*_1,B _= 491/1092, 226/509; ΔLL = 0.022;*P *= 0.833. Assuming DY_D _and M have the same underlying sex ratio (MLEs 0.44, 0.45), and comparing them to DY_M_, we have DY_M_|{DY_D_, M}*p*_0 _= 1262/2663; *p*_1,A_, *p*_1,B _= 545/1062, 717/1601; ΔLL = 5.466; *P *= 0.001.

## Discussion and conclusion

In this paper, we demonstrate two striking observations with regard to the Czech-Bavarian mouse contact zone: (1) a large geographic anomaly with respect to the *musculus *Y chromosome; and (2) a significant difference in the census sex ratio in those *domesticus *localities where the *musculus *Y chromosome is present, compared to both *musculus *localities and *domesticus *localities without Y introgression. We suggest it is unlikely that these two patterns are coincidental. We discuss each observation in turn, and then explore whether a single cause might parsimoniously explain both.

### Geographic anomaly of Y introgression

We present strong evidence that the *M. m. musculus *Y chromosome is introgressed on the *M. m. domesticus *side of the Czech-Bavarian contact zone between the two house mouse subspecies, penetrating some 22 km and over an extensive region of approximately 330 km^2^. The introgression is shown particularly clearly when monotonic clines are fitted to the data at multiple loci (Figure 4a): all clines save the Y chromosome change abruptly across an interval of less than one kilometre, i.e., less than the estimated scale of movement of mice per generation [14,23]. It may be that monotonic cline hypotheses, which allow discrete jumps in allele frequencies between localities, are particularly suited to describing the demic nature of house mouse population density. Compared to the strong coincidence of cline centres over eight loci, the non-coincidence of the Y chromosome is striking.

Another notable characteristic of the Y chromosome transition is its width (when assessed for the correct orientation), which is comparable to other portions of the hybrid zone. This might seem surprising because the Y has escaped linkage disequilibrium with other loci at the centre of the consensus zone. Such association is expected to steepen clines [12] thus we might expect the escapee Y cline to be wider than in portions of the hybrid zone where it coincides with the consensus. However, while a narrow cline will tend to steepen wider associated clines, we do not expect the reverse to the same degree – a wide cline is likely to have little effect in steeping an associated cline that already changes at a much smaller scale. In other words, the consistent width of the Y indicates it is likely to be a *driver *of the steep change at other loci when in coincidence at secondary contact rather than a *follower *(through statistical association) of action at some other, as yet undetected, locus. This is perhaps unsurprising given evidence that the differentiation of the Y-chromosome lineages may contribute to partial reproductive isolation between *Mus *subspecies [33].

Two contrasting scenarios could equally explain the current geographic pattern of the Y chromosome introgression: (A) a static consensus centre and westward geographic incursion of the *musculus *Y chromosome, forming a bulge or *salient*; and (B) a dynamic consensus centre that has moved eastwards, leaving an *invagination *of the *musculus *Y chromosome in its wake. These salient/invagination scenarios are not mutually exclusive: both the consensus centre and the *musculus *Y front may have moved. The major evolutionary implication is, however, unaffected by these details: in the current introgression region, the *musculus *Y chromosome has obviously been successful relative to the *domesticus *Y chromosome. This is clear if we assume the *musculus *Y has pushed west through the consensus centre, replacing the *domesticus *Y on its own background, and forming a salient. However, it is also true if the consensus centre has moved east: because the coincidence of clines is expected to be maintained by a tension zone, we expect the *domesticus *Y to advance east along with the consensus centre. If an invagination of *musculus *Y chromosomes is formed during this advance, it is because they have resisted the movement of the consensus centre, again replacing *domesticus *Y chromosomes on their own background. Thus the evolutionary implications are the same irrespective of how the displacement between the Y front and the consensus centre came about. It is their *relative *positions which inform us that the *musculus *Y has been successful in comparison with the *domesticus *Y.

The *musculus *Y chromosome introgression is surprising for two reasons, one specific to the house mouse zone, and one more general. First, *domesticus *males are known to be more aggressive than *musculus *males [34-36], and given the male-dominated harem mating system of house mice, we might expect the *domesticus *Y to have an advantage over the *musculus *Y where the two subspecies meet. Second, and more generally, Haldane's rule [37] predicts that it is the heterogametic sex that is preferentially affected by hybrid incompatibilities. When the heterogametic sex is female, mitochondrial transmission between diverged populations should be impeded: as pointed out by Mallet [38] this may explain why mitochondrial introgression is more common in mammals and flies than in birds and bees [39]. Britton-Davidian et al. [40] demonstrated that Haldane's rule holds for hybrids between *musculus *and *domesticus *mice from Denmark: male F1's are sterile and so we might expect Y chromosome transmission between *musculus *and *domesticus *to be impeded because Y chromosomes can only be transmitted through the heterogametic sex, i.e., males. The sharp and coincident Y chromosome clines seen elsewhere in the European house mouse hybrid zone are also consistent with this argument.

The above reasons add to the degree of surprise on observing the *musculus *Y introgression, but they may be balanced against other lines of evidence. First, it has been shown that both *musculus *males and *musculus *females prefer individuals of the same subspecies while *domesticus *mice are not choosy [36,41-47], though homosubspecific preference has also been detected in *domesticus *individuals in some populations in Denmark and central Europe (G. Ganem and B. Bímová, unpubl. results). All other things being equal, reduced choosiness in *domesticus *should favour introgression of alleles from *musculus *into *domesticus*, relative to the reverse. This is not an argument for selection favouring introgression, rather for a barrier to neutral introgression (choosiness) being removed in one direction. Reduced choosiness would only confer an invasive advantage to *musculus *alleles if finding a mate were difficult. In our study area, this situation seems unlikely since there was little difficulty trapping mice of both sexes. Furthermore, a neutral asymmetry in prezygotic barriers seems unlikely to give rise to the observed salient of Y introgression since its extent is large relative to the movement of mice and its front is sharp: to be large and neutral, the introgression process would have to be old, and an old stochastic process would not exhibit a sharp front. Finally, it should be noted that mice toward the centre of the hybrid zone are the descendants of many generations of intercrossing making it unlikely for their mate choice behaviour to be either pure *musculus*-like or pure *domesticus*-like.

Second, although Britton-Davidian et al. [40] demonstrate Haldane's rule for *musculus*-*domesticus *hybrids, male F1's being infertile, the same study reveals that the homogametic sex may also be affected: females appear significantly underrepresented in the sex ratio at birth in crosses between Danish *musculus *and *domesticus *(in total, 231 males : 180 females; sex ratio *p *= 0.562; Pr(Bin) = 0.007). The significance of this bias in the birth sex ratio was not noted at the time of the study because results were assessed relative to the χ^2 ^distribution, which only approximates to comparison with the binomial distribution if numbers are large. If only males were adversely affected in the F1 generation, Y introgression might be particularly surprising. This crossing data suggests, however, that both sexes are adversely affected, although in different ways. Third, crosses between inbred Czech *musculus *and inbred French *domesticus*, described in the same study [40], revealed that F1's fathered by Czech males were not infertile. These crosses may have involved genetic backgrounds more similar to the current Czech-Bavarian contact than the Danish crosses, and raise the possibility of a unidirectional breakdown of Haldane's rule facilitating *musculus *Y introgression.

### Sex ratio anomaly in localities with Y introgression

We demonstrate that when localities with Y introgression are excluded the census sex ratio in both the *domesticus *and *musculus *regions of the field area is significantly, and similarly, female biased. In contrast, the sex ratio in localities with Y introgression is close to parity and significantly different from both *musculus *and non-introgressed *domesticus *(Table 2). Two potential sources of uncertainty exist in the sex ratio measurements. First, genetic studies of sex determination in laboratory mice have shown that the sex determining pathway is sensitive to genetic background [48-51]. As the *musculus *Y is introgressed onto a new background, we must address the possibility that the associated sex ratio perturbation is phenotypic, but not genetic, and caused by disruption of the sex determination pathway. However, we can discount this possibility because 1) the majority of individuals were typed both phenotypically and genetically, with a mismatch rate of less than 1%, consistent simply with a very low rate of human error; 2) if the *musculus *Y on the *domesticus *background did indeed cause developmental sex reversal, XY individuals being phenotypically females [48-51], then we should expect a relative *excess *of phenotypic females in introgressed localities, and not the observed relative female *deficit*. Second, the propensity of females *versus *males to enter traps is unknown and so we do not know if the census sex ratio is an unbiased reflection of the tertiary sex ratio. However, in a similar fashion to the comparison of *relative *cline positions above, the *relative *census sex ratios are revealing. If the trapping propensity of sexes is similar throughout the field area, it is clear that the tertiary sex ratio in introgressed localities has a higher proportion of males than other localities. It is possible that Y introgression changes the behaviour of males, making them more likely to enter traps. However, this would represent elevated risk-taking behaviour. It should be remembered that the loss of mice from the population due to once-yearly trapping by scientists is likely negligible compared to other losses. Then, elevated risk-taking behaviour should result in a *deficit *of males in our samples, because of the (fatal) risks they have taken throughout the year, rather than the observed relative male excess. The elevated proportion of males trapped in introgressed localities is therefore unlikely to be due to a change in male behaviour and we conclude that it is the tertiary sex ratio itself that in introgressed localities is composed of a higher proportion of males than other localities.

The estimated sex ratio varies among localities and it is only the strength of the effect in introgressed localities combined with massive sampling effort across more than 120 localities in total that allows a significant difference between locality categories to be revealed. Variation in census sex ratio between localities might be expected not only because of the stochastic nature of finite samples: for example, Rosenfeld et al. [52] show that female mice bias the secondary sex ratio of their offspring as a function of their diet. Food availability of commensal mice will vary naturally from farm to farm across the field area, and from year to year. Similarly, parasite prevalence and load is typically highly spatiotemporally heterogeneous, and Kaòková et al. [53] demonstrate perturbation of the secondary sex ratio during the latent phase of toxoplasmosis in mice. For such reasons it is probably unhelpful to attempt to reason in terms of a singular evolutionarily stable sex ratio in wild mouse populations. The observed sex ratio will arise as a function of local resource availability and the interaction of numerous competing strategies for investing those resources and maximising evolutionary gains. The commonality of these strategies is that each is played out to some extent through favouring numbers of individuals of one sex over the other. Where parasite genes benefit from such a strategy, the interaction is usually called host manipulation [53], where particular mouse genes benefit, genetic conflict [54,55], while in the absence of any such manipulation/conflict, strategy at the level of parental individuals is shaped only by natural selection [56]. A recent study (J. Piálek, unpubl. results) shows that significant parent-specific variation in the outcome of such strategies can be demonstrated by measuring secondary sex ratio over multiple litters. Of 258 wild caught mice pairs taken from 29 populations across the Czech-Bavarian portion of the zone and crossed in the laboratory under uniform intermediate nutrition levels, one pair from the *musculus *region has shown significant male bias (104 males, 76 females, sex ratio *p *= 0.58, Pr(Bin) = 0.022). It would be interesting to see what further variation might be revealed under high and low nutrition regimes and parasite loads.

### Reconciling the Y chromosome geographic and sex ratio patterns

The geographic pattern indicates the *musculus *Y chromosome has been successful relative to the *domesticus *Y on the *domesticus *background. The sex ratio pattern indicates that *domesticus *localities with *musculus *Y introgression have an elevated proportion of males in the tertiary sex ratio. A parsimonious explanation of both observations is that the success of the introgressing Y chromosome is *because *the combination of the Y and its novel genetic background increases the proportion of fertile males. As a result, where both *domesticus *and *musculus *Y chromosomes are present, the *musculus *Y tends to replace the *domesticus *alternative. An interesting question then arises: is this tendency acting in the same direction as natural selection or against the action of natural selection? The answer depends on whether, in the absence of Y introgression, the populations would be at equilibrium sex ratio regarding investment in the sexes [57], or biased in favour of females by selfish elements. In the first case, the action of the introgressing Y is selfish, i.e., in conflict with natural selection. In the second case the interests of the introgressing Y chromosome are aligned with natural selection, and its success may be speeded as it is redressing an imbalance caused by other (selfish) elements. In either case, selfish elements, and therefore genetic conflicts, are implicated. Deciding which of the alternative contexts is more likely requires information about potential differential investment in the sexes, of which little has been published. Male and female birth weights are very similar in these mice (J. Piálek, unpubl. results) and while it seems males may demand more resources during lactation, no female sex ratio bias has been reported in other areas of the mouse contact front, despite large sample sizes. These observations would suggest that differential investment in the sexes is in general not sufficient for the mouse sex ratio to differ significantly from parity. Then, the marked female trapping bias in the Czech-Bavarian zone observed outside of introgressed localities may indicate the presence of female favouring distortion elements, and the introgressing Y chromosome would be redressing a female imbalance in the sex ratio and thus be acting in the same direction as natural selection. In this case we might seek evidence of the previous spread of a selfish female biasing factor.

The similarity between the geographic pattern of the Y chromosome introgression and the *musculus *mitochondrion might seem unremarkable – once a barrier to gene flow is broken down, perhaps many elements might flow through. On reflection, however, the mitochondrial pattern is perplexing, because the mitochondrial genome and Y chromosome are never (or at most, extremely rarely) co-inherited, and so *musculus *mitochondrial variants quite simply could not have hitch-hiked through statistical association during the selective success of the introgressing Y. Furthermore, the interests of maternally and paternally inherited elements are in conflict with respect to the sex ratio, making it very unlikely that the mitochondrial and Y chromosome geographic patterns arose simultaneously. We are forced to conclude that one followed the other without statistical association, yet ending up covering a similar area of the field. Their conflicting interests over the sex ratio could explain this conundrum – the mitochondrion *would *hitch-hike with a *female *biasing distorter of the sex ratio, and an introgressing Y which rescues that distortion would then be favoured by natural selection, wherever the distorter had passed.

Irrespective of speculation regarding the direction of interests, selfish or otherwise, of the *musculus *Y chromosome, if the increased proportion of males in introgressed localities is the cause of the *musculus *Y success, then this is the first case where genetic conflict has been implicated as a force causing the decay of a species barrier. Taking other transects of the mouse contact hybrid zone in Europe as representative of the Czech-Bavarian contact before the Y incursion, originally there were sharp coincident changes for sex chromosome markers, and centred on these more gradual clines at autosomal loci: the actions of many barrier loci were focused in a single geographic region. Now, the Y chromosome cline has escaped the forces holding these barriers in coincidence and the total effective barrier in that region is reduced: no change in Y chromosome state occurs across the consensus hybrid zone centre in the southern half of our field area.

In the Introduction we noted that there is growing evidence that the sex chromosomes harbour more genes causing disruption of fertility and/or viability in hybrids than autosomes, and that while the causes of this disruption have generally been assumed to follow the Dobzhansky-Muller model of accumulation of incompatibilities [4], there is also increasing evidence that genes involved in genetic conflicts may be implicated [9-11,58,59]. We stated that irrespective of the causes of such disruption, gene flow of sex linked markers across a hybrid zone is expected to be impeded resulting in abrupt clines [12]. In the current study we show an abrupt Y chromosome cline has escaped the consensus centre of the mouse hybrid zone, and that Y introgression is associated with a significant perturbation in the tertiary sex ratio towards males. Either these two striking patterns are coincidental, or the results of genetic conflict are currently laid out across the landscape of the hybrid zone, having overcome the selective forces of heterogametic incompatibility that focus genetic change into a single geographic region.

## Methods

### Sampling

In total, 2311 house mice were trapped at 126 sites scattered across a belt 145 km long and 50 km wide, stretched from north-eastern Bavaria (Germany) to western Bohemia (Czech Republic). The sampling sites are listed in the Appendix and their position is shown in Fig 1. All trapped mice were euthanized and dissected either in a field laboratory or in the Studenec laboratory and their sex and reproductive status was inspected immediately after dissection.

Since sampled alleles (see below) may be non-independent due to relatedness and deviations from Hardy-Weinberg equilibrium, we used the effective number of alleles calculated according to [60] and [23]:

(1) $$N_{\text{e}}=\frac{2N}{2NF_{\text{ST}}+\left(1-F_{\text{ST}}\right)\left(1+\left|F_{\text{IS}}\right|\right)},$$

where *N *is the number of diploid individuals sampled at a site, *F*_IS _is the measure of deficit/excess of heterozygotes, and *F*_ST _represents the measure of relatedness in each population (note that the term (1 - *F*_ST_) disappeared from the original formula as a typing error in [60], a mutation propagated in [23]). This means that if all sampled individuals were completely related, the effective sample size would be 1 and, conversely, if individuals were completely unrelated, this would result in *N*_e _ranging from *N *(when *F*_IS _= 1) to 2*N *(when *F*_IS _= 0). Thus for high *F*_ST _values the influence of large samples is substantially reduced. For haploid markers *F*_IS _= 0 and so the formula (1) simplifies to:

(2) $$N_{\text{e}}=\frac{N}{NF_{\text{ST}}+1-F_{\text{ST}}}.$$

In the case of the X chromosome, estimation of *N*_e _is slightly more complex because the chromosome is diploid in females and haploid in males. If *N*_m _and *N*_f _are the number of sampled males and females, respectively, and $F_{{\text{IS}}_{\text{f}}}$ is the measure of deficit/excess of heterozygotes in females, then the effective number of alleles is given as:

(3) $$N_{\text{e}}=\frac{2N_{\text{f}}+N_{\text{m}}\left(1+\left|F_{{\text{IS}}_{\text{f}}}\right|\right)}{2N_{\text{f}}F_{\text{ST}}+\left[1-F_{\text{ST}}\left(1-N_{\text{m}}\right)\right]\left(1+\left|F_{{\text{IS}}_{\text{f}}}\right|\right)}.$$

*F*_IS _was estimated for each marker and for each population separately, whereas for *F*_ST _the maximum likelihood estimate (MLE) was calculated for each population as a combined estimate from genotypes at all loci (see below) using both the Beta and normal distribution options from Analyse 1.3 [61]. The consensus* orientation estimates were similar irrespective of the model used for *F*_ST _estimation. Here we show only results for the Beta distribution option since this results in higher estimates of *F*_ST _and, hence, is more conservative with regard to effective sample sizes.

An estimation of *F*_IS _is problematic in some extreme cases. For example, if one of the classes (*pp*, *pq*, *qq*) contains a zero element and the second one is close to zero, then MLE of *F*_IS _will be 1 (e.g., when *pp *= 50, *pq *= 0, *qq *= 1) or -1 (e.g., when *pp *= 0, *pq *= 1, *qq *= 50) irrespective of the "true" value. So we used the following rule of thumb: if one of the counts was zero and the second lowest count was less than 3, then we assumed we had not enough information to justify an *F*_IS _deviation from zero. Even though this choice is obviously arbitrary the resulting estimates are rather conservative and the number of outliers is reduced.

### Markers

DNA was isolated from tissues using proteinase K digestion and subsequent extraction either with phenol-chloroform and ethanol precipitation [61] or using the DNeasy^® ^96 Tissue Kit (QIAGEN), following standard protocols or the manufacturer's instructions.

Males (*N *= 1050; 115 sites) were typed for the presence or absence of an 18-bp deletion, located within the last exon of the Zinc finger protein 2, Y linked, gene (*Zfy2*) using the method given in [62]. The deletion is fixed in *M. m. musculus *and absent in *M. m. domesticus *[23,63]. In addition, the results were verified using a Y-specific microsatellite (TTTTG repeat) at the 5'-end of the second intron of *Zfy2 *under the same conditions as described in [23]. Since there was no incongruence between the two Y-chromosome markers, they are henceforth referred to as a single marker.

To set the Y chromosome results within the context of the hybrid zone, we used one X-linked and seven autosomal markers. For the X-chromosome marker, we scored a B1 SINE retroelement, mapping the *Btk *gene (*N *= 2171; 126 sites): this insertion is present in *M. m. domesticus *and absent in *M. m. musculus *(see [23,64] for details). For autosomal markers, six allozyme loci (*Es1*, *Gpd1*, *Idh1*, *Mpi*, *Np*, *Sod1*; *N *= 1965–2137; 126 sites) were used as described in [14]. The seventh autosomal marker maps the *Abpa *gene coding for the alpha-subunit of the salivary Androgen binding protein (Chromosome 7; *N *= 2056; 126 sites) following [22].

Finally, we compared the data with the distribution of a mitochondrial DNA marker across the area under study. This marker was represented by the *Bam*HI restriction site mapped at position 3565 of the standard mouse mtDNA sequence [65] which has been shown to be a reliable marker distinguishing the two house mouse subspecies [66,67]. 1939 specimens from 121 sites were analyzed as described in [67].

### Analyses

The orientation of change in allele frequencies in two-dimensional space was estimated with the Pooled Adjacent Violators Algorithm (PAVA), originally described by Brunk [68] as a method for finding the maximum likelihood monotonic cline over a set of observations (see also [69,70]). One advantage of this method is that it is not dependent on any particular cline model: PAVA assumes no particular shape of change other than monotonic increase or decrease across a series of sampling sites. For this reason, the distance between localities does not affect the outcome of PAVA, only their ordering. An ordering is found by taking the orthogonal projection of localities onto a cross-section through the field area, and then noting the order in which the projected localities fall along this cross-section. An implicit assumption is that at the scale of observation the path of the contact front is linear. For a finite number *N *of sampling localities there are ≤ *N*(*N *– 1) distinct orderings so a complete likelihood profile for monotonic change across the continuous interval of orientations [0°,360°] can be calculated. No sampling or search algorithms need be applied. Likelihood profiles and their underlying monotonic cline estimates were calculated for each marker using a routine written in Mathematica [71]. In order to test whether orientations were similar across loci, we compared likelihood profiles as described in [14] and [58]. The width of a cline is normally measured as the inverse of the maximum gradient. It is not clear how best to apply this metric to PAVA monotonic clines, so widths were estimated by fitting sigmoid or stepped models using the Analyse program [60].

## Authors' contributions

MM, PM, PD, BB, JP collected the material and MM, PM, and JP participated in the design of the study. MM analyzed allozyme markers and participated in the analysis of the microsatellite locus. PM, PD, BB performed laboratory experiments except those carried out by MM. JP provided unpublished data on sex ratio from breeding experiments and logistical support for the study. SJEB analyzed the data, interpreted the results and wrote the paper as it arose out of intensive discussion with MM. All authors read and approved the final version of the manuscript.

## Supplementary Material

Additional file 1 **A list of sampling sites**. For each site, geographic coordinates (Latitude, Longitude), category (M = *musculus *population; DyM = *domesticus *population with *musculus *Y introgressed; DyD = *domesticus *population with no introgressed *musculus *Y), total number of individuals and the number of males trapped, and the total number of alleles and the number of *domesticus *alleles for each marker (mtDNA, Y chromosome, *Btk*, *Abpa*, *Es1*, *Gpd1*, *Idh1*, *Mpi*, *Np*, *Sod1*) are given. Click here for file

## References

[B1] Grula JW Taylor OR Some characteristics of hybrids derived from the sulphur butterflies, *Colias eurytheme *and *Colias philodice*: phenotypic effects of the X chromosome Evolution 1980 34 673 687 10.2307/2408021 28563988

[B2] Zouros E Lofdahl K Martin PA Male hybrid sterility in *Drosophila*: interactions between autosomes and sex chromosomes in crosses of *D*. *mojavensis *and *D. arizonensis* Evolution 1988 42 1321 1331 10.2307/2409015 28581068

[B3] Coyne JA Orr HA Otte D, Endler J Two rules of speciation Speciation and its Consequences 1989 Sunderland, MA: Sinauer Associates, Inc 180 207

[B4] Coyne JA Orr HA Speciation 2004 Sunderland, MA: Sinauer Associates, Inc

[B5] Prowell DP Howard DJ, Berlocher SH Sex linkage and speciation in Lepidoptera Endless Forms: Species and Speciation 1998 Oxford: Oxford University Press 309 319

[B6] Jiggins CD Linares M Naisbit RE Salazar C Yang ZH Mallet J Sex-linked hybrid sterility in a butterfly Evolution 2001 55 1631 1638 11580022 10.1111/j.0014-3820.2001.tb00682.x

[B7] Tao Y Chen S Hartl DL Laurie CC Genetic dissection of hybrid incompatibilities between *Drosophila simulans *and *D. mauritiana*. I. Differential accumulation of hybrid male sterility effects on the X and autosomes Genetics 2003 164 1383 1397 12930747 10.1093/genetics/164.4.1383 PMC1462656

[B8] Counterman BA Ortíz-Barrientos D Noor MAF Using comparative genomic data to test for fast-X evolution Evolution 2004 58 656 660 15119449

[B9] Tao Y Hartl DL Laurie CC Sex-segregation distortion associated with reproductive isolation in *Drosophila* Proc Natl Acad Sci USA 2001 98 13183 13188 10.1073/pnas.231478798 11687638 PMC60845

[B10] Orr HA Irving S Segregation distortion in hybrids between the Bogota and USA subspecies of *Drosophila pseudoobscura* Genetics 2005 169 671 682 10.1534/genetics.104.033274 15654115 PMC1449097

[B11] Orr HA Masly JP Phadnis N Speciation in *Drosophila*: From phenotypes to molecules J Hered 2007 98 103 110 10.1093/jhered/esl060 17194791

[B12] Barton NH Gale KS Harrison RG Genetic analysis of hybrid zones Hybrid Zones and the Evolutionary Process 1993 Oxford: Oxford University Press 13 45

[B13] Boursot P Auffray J-C Britton-Davidian J Bonhomme F The evolution of house mice Annu Rev Ecol Syst 1993 24 119 152 10.1146/annurev.es.24.110193.001003

[B14] Macholán M Munclinger P Šugerková M Dufková P Bímová B Božíková E Zima J Piálek J Genetic analysis of autosomal and X-linked markers across a mouse hybrid zone Evolution 2007 61 746 771 10.1111/j.1558-5646.2007.00065.x 17439609

[B15] Vanlerberghe F Boursot P Catalan J Guerasimov S Bonhomme F Botev B Thaler L Analyse génétique de la zone d'hybridation entre les deux sous-espèces de souris *M*. *m. domesticus *et *M. m. musculus *en Bulgarie Genome 1988 30 427 437 3169546

[B16] Tucker PK Sage RD Warner J Wilson AC Eicher EM Abrupt cline for sex chromosomes in a hybrid zone between two species of mice Evolution 1992 46 1146 1163 10.2307/2409762 28564395

[B17] Payseur BA Krenz JG Nachman MW Differential patterns of introgression across the X chromosome in a hybrid zone between two species of house mice Evolution 2004 58 2064 2078 15521462 10.1111/j.0014-3820.2004.tb00490.x

[B18] Dod B Jermiin LS Boursot P Chapman VH Tonnes-Nielsen J Bonhomme F Counterselection on sex chromosomes in the *Mus musculus *European hybrid zone J Evol Biol 1993 6 529 546 10.1046/j.1420-9101.1993.6040529.x

[B19] Vanlerberghe F Boursot P Nielsen JT Bonhomme F A steep cline for mitochondrial DNA in Danish mice Genet Res 1988 52 185 193 3243422 10.1017/s0016672300027646

[B20] Prager EM Boursot P Sage RD New assays for Y chromosome and p53 pseudogene clines among East Holstein house mice Mamm Genome 1997 8 279 281 10.1007/s003359900410 9096111

[B21] Vanlerberghe F Dod B Boursot P Bellis M Bonhomme F Absence of Y-chromosome introgression across the hybrid zone between *Mus musculus domesticus *and *Mus musculus musculus* Genet Res 1986 48 191 197 3569903 10.1017/s0016672300025003

[B22] Dod B Smadja C Karn RC Boursot P Testing for selection on the androgen-binding protein in the Danish mouse hybrid zone Biol J Linn Soc 2005 84 447 459 10.1111/j.1095-8312.2005.00446.x

[B23] Raufaste N Orth A Belkhir K Senet D Smadja C Baird SJE Bonhomme F Dod B Boursot P Inferences of selection and migration in the Danish house mouse hybrid zone Biol J Linn Soc 2005 84 593 616 10.1111/j.1095-8312.2005.00457.x

[B24] Munclinger P Božíková E Šugerková M Piálek J Macholán M Genetic variation in house mice (*Mus*, Muridae, Rodentia) from the Czech and Slovak Republics Folia Zool 2002 51 81 92

[B25] Auffray J-C Vanlerberghe F Britton-Davidian J The house mouse progression in Eurasia: a palaeontological and archaeozoological approach Biol J Linn Soc 1990 41 13 25 10.1111/j.1095-8312.1990.tb00818.x

[B26] Sage RD Atchley WR Capanna E House mice as models in systematic biology Syst Biol 1993 42 523 561 10.2307/2992487

[B27] Cucchi T Vigne J-D Auffray J-C First occurrence of the house mouse(*Mus musculus domesticus *Schwartz & Schwartz, 1943) in the western Mediterranean: a zooarchaeological revision of sub-fossil house mouse occurrences Biol J Linn Soc 2005 84 429 445 10.1111/j.1095-8312.2005.00445.x

[B28] Bazykin AD Hypothetical mechanism of speciation Evolution 1969 23 685 687 10.2307/2406862 28562864

[B29] Ferris C Rubio JM Gosalvez J Hewitt GM One-way introgression of subspecific sex-chromosome marker in a hybrid zone Heredity 1993 71 119 129 10.1038/hdy.1993.115

[B30] Searle JB Wójcik JM Wójcik JM, Wolsan M Chromosomal evolution: The case of *Sorex araneus* Evolution of Shrews 1998 Bialowieza: Mammal Research Institute, Polish Academy of Sciences 219 268

[B31] Bridle JR Baird SJE Butlin RK Spatial structure and habitat variation in a grasshopper hybrid zone Evolution 2001 55 1832 1843 11681738 10.1111/j.0014-3820.2001.tb00832.x

[B32] MacCallum CJ Nürnberger B Barton NH Szymura JM Habitat preference in the *Bombina *hybrid zone in Croatia Evolution 1998 52 227 239 10.2307/2410938 28568140

[B33] Boissinot S Boursot P Discordant phylogeographic patterns between the Y chromosome and mitochondrial DNA in the house mouse: Selection on the Y chromosome? Genetics 1997 146 1019 1034 9215905 10.1093/genetics/146.3.1019 PMC1208032

[B34] Thuesen P A comparison of the agonistic behaviour of the *Mus musculus musculus *L. and *Mus musculus domesticus *Rutty (Mammalia and Rodentia) Vidensk Dan Naturhist Foren 1977 140 117 128

[B35] van Zegeren K van Oortmerssen GA Frontier disputes between the West and East-European house mouse in Schleswig-Holstein, West Germany Z Säugetierkd 1981 46 363 369

[B36] Piálek J Vyskočilová M Bímová B Havelková D Piálková J Dufková P Bencová V Ďureje L' Albrecht T Hauffe HC Macholán M Munclinger P Storchová R Zajícová A Holáň V Gregorová S Forejt J Development of unique house mouse resources suitable for evolutionary studies of speciation J Hered 2008 99 1 34 44 10.1093/jhered/esm083 17965200

[B37] Haldane JBS Sex ratio and unisexual sterility in hybrid animals J Genet 1922 12 101 109

[B38] Mallet J Hybridization as an invasion of the genome Trends Ecol Evol 2005 20 229 237 10.1016/j.tree.2005.02.010 16701374

[B39] Sperling FAH Natural hybrids of *Papilio *(Insecta: Lepidoptera): poor taxonomy or interesting evolutionary problem? Can J Zool 1990 68 790 1799 10.1139/z90-260

[B40] Britton-Davidian J Fel-Clair F Lopez J Alibert P Boursot P Postzygotic isolation between the two European subspecies of the house mouse: estimates from fertility patterns in wild and laboratory-bred hybrids Biol J Linn Soc 2005 84 379 393 10.1111/j.1095-8312.2005.00441.x

[B41] Munclinger P Frynta D Social interactions within and between two distant populations of house mouse Folia Zool 1997 46 193 199

[B42] Christophe N Baudoin C Olfactory preferences in two subspecies of mice *Mus musculus musculus *and *Mus musculus domesticus *and their hybrids Anim Behav 1998 56 365 369 10.1006/anbe.1998.0798 9787027

[B43] Smadja C Ganem G Subspecies recognition in the house mouse: a study of two populations from the border of a hybrid zone Behav Ecol 2002 13 312 320 10.1093/beheco/13.3.312

[B44] Smadja C Ganem G Asymmetrical reproductive character displacement in the house mouse J Evol Biol 2005 18 1485 1493 16313461 10.1111/j.1420-9101.2005.00944.x

[B45] Smadja C Catalan J Ganem G Strong premating divergence in a unimodal hybrid zone between two subspecies in the house mouse J Evol Biol 2004 17 165 176 10.1046/j.1420-9101.2003.00647.x 15000659

[B46] Bímová B Karn RC Piálek J The role of salivary androgen-binding protein in reproductive isolation between two subspecies of house mouse: *Mus musculus musculus *and *Mus musculus domesticus* Biol J Linn Soc 2005 84 349 361 10.1111/j.1095-8312.2005.00439.x

[B47] Frynta D Slábová M Třeštíková H Volfová R Munclinger P Aggression and commensalism in house mouse: a comparative study across Europe and Near East Aggressive Behav 2005 31 283 293 10.1002/ab.15555

[B48] Eicher EM Washburn LL Genetic control of sex determination in mice Annu Rev Genet 1986 20 327 360 10.1146/annurev.ge.20.120186.001551 3545061

[B49] Eicher EM Washburn LL Schork NJ Lee BK Shown EP Xu X Dredge RD Pringle MJ Page DC Sex-determining genes on mouse autosomes identified by linkage analysis of C57BL/6J-YPOS sex reversal Nat Genet 1996 14 206 209 10.1038/ng1096-206 8841197

[B50] Washburn LL Eicher EM Sex reversal in XY mice caused by dominant mutation on chromosome 17 Nature 1983 303 338 340 10.1038/303338a0 6855886

[B51] Washburn LL Albrecht KH Eicher EM C57BL/6J- *T*-associated sex reversal in mice is caused by reduced expression of a *Mus domesticus Sry *allele Genetics 2001 158 1675 1681 11514455 10.1093/genetics/158.4.1675 PMC1461743

[B52] Rosenfeld CS Grimm KM Livingston KA Brokman AM Lamberson WE Roberts RM Striking variation in the sex ratio of pups born to mice according to whether maternal diet is high in fat or carbohydrate Proc Nat Acad Sci USA 2003 100 4628 4632 10.1073/pnas.0330808100 12672968 PMC153606

[B53] Kaňková Š Kodym P Frynta D Vavřinová R Kuběna A Flegr J Influence of latent toxoplasmosis on the secondary sex ratio in mice Parasitology 2007 134 1709 1717 17651529 10.1017/S0031182007003253

[B54] Werren JH Beukeboom LW Sex determination, sex ratios, and genetic conflict Annu Rev Ecol Syst 1998 29 233 261 10.1146/annurev.ecolsys.29.1.233

[B55] Burt A Trivers R Genes in Conflict: The Biology of Selfish Genetic Elements 2006 Cambridge, MA: Harvard University Press

[B56] Trivers RL Willard DE Natural-selection of parental ability to vary sex-ratio of offspring Science 1973 179 90 92 10.1126/science.179.4068.90 4682135

[B57] Fisher RA The Genetical Theory of Natural Selection 1930 Oxford: Oxford University Press

[B58] Frank SA Divergence of meiotic drive-suppression systems as an explanation for sex-biased hybrid sterility and inviability Evolution 1991 45 262 267 10.2307/2409661 28567880

[B59] Hurst LD Pomiankowski A Causes of sex ratio bias may account for unisexual sterility in hybrids: a new explanation of Haldane's rule and related phenomena Genetics 1991 128 841 858 1916248 10.1093/genetics/128.4.841 PMC1204557

[B60] Phillips BL Baird SJE Moritz C When vicars meet: a narrow contact zone between morphologically cryptic phylogeographic lineages of the rainforest skink, *Carlia rubrigularis* Evolution 2004 58 1536 1548 15341156 10.1111/j.0014-3820.2004.tb01734.x

[B61] Barton NH Baird SJE Analyse – An application for analyzing hybrid zones Available at request from Stuart JE Baird 1995 Freeware, Edinburgh, UK

[B62] Hoelzel AR Green A Hoelzel AR Analysis of population-level variation by sequencing PCR-amplified DNA Molecular Genetic Analysis of Populations. A Practical Approach 1992 New York: Oxford University Press 159 280

[B63] Nagamine CM Nishioka Y Moriwaki K Boursot P Bonhomme F Lan YFC The *musculus *type Y chromosome of the laboratory mouse is of Asian origin Mamm Genome 1992 3 84 91 10.1007/BF00431251 1352158

[B64] Munclinger P Boursot P Dod B B1 insertions as easy markers for mouse population studies Mamm Genome 2003 14 359 366 10.1007/s00335-002-3065-7 12879357

[B65] Bibb MJ van Etten RA Wright CT Walberg MW Clayton DA Sequence and gene organization of mouse mitochondrial DNA Cell 1981 26 167 180 10.1016/0092-8674(81)90300-7 7332926

[B66] Boursot P Din W Anand R Darviche D Dod B Deimling FV Talwar GP Bonhomme F Origin and radiation of the house mouse: mitochondrial DNA phylogeny J Evol Biol 1996 9 391 415 10.1046/j.1420-9101.1996.9040391.x

[B67] Božíková E Munclinger P Teeter KC Tucker PK Macholán M Piálek J Mitochondrial DNA in the hybrid zone between *Mus musculus musculus *and *Mus musculus domesticus*: a comparison of two transects Biol J Linn Soc 2005 84 363 378 10.1111/j.1095-8312.2005.00440.x

[B68] Brunk HD Maximum likelihood estimates of monotone parameters Ann Math Stat 1955 26 607 616 10.1214/aoms/1177728420

[B69] Barlow RE Statistical Inference under Order Restrictions: The Theory and Application of Isotonic Regression 1972 London: John Wiley and Sons

[B70] Johnson T Multipoint linkage disequilibrium mapping using multilocus allele frequency data Ann Hum Genet 2005 69 474 497 10.1046/j.1529-8817.2005.00178.x 15996175

[B71] Wolfram S Mathematica: A System for Doing Mathematics by Computer 1992 Redwood City: Addison-Wesley Publishing Company, Inc

